# About Dice, Bouldering, and Team Empowerment: Running the CompOmics Group at VIB and Ghent University, Belgium

**DOI:** 10.1371/journal.pcbi.1003332

**Published:** 2013-11-07

**Authors:** Lennart Martens

**Affiliations:** 1 Department of Medical Protein Research, VIB, Ghent, Belgium; 2 Department of Biochemistry, Ghent University, Ghent, Belgium; University of Bremen, Germany

## Introduction[Fig pcbi-1003332-g001]

**Image 1 pcbi-1003332-g001:**
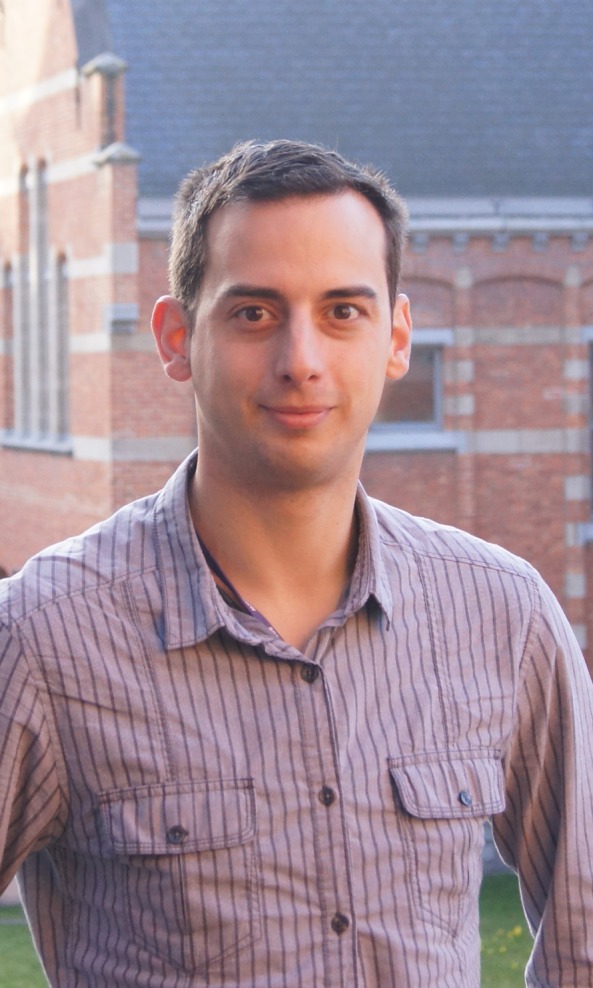
Photo of Lennart Martens. Prof. Martens has been leading the Computational Omics and Systems Biology (CompOmics) Group at VIB and Ghent University, both in Ghent, Belgium, since October 2009.

Starting up a new lab is of a course a great challenge, but few young PIs will realize that getting started is the easy part. Indeed, once the lab is up and running, and hopefully doing very well, a whole new set of challenges emerges on top of the typical ones like fund raising and paper publication. Perhaps most dauntingly, you'll be expected to manage the different people in your group, building on and developing the strengths of each individual while simultaneously forging a close-knit team that can collectively tackle the toughest tasks you give them. Obviously, most scientists, including me, are poorly trained for management, and figuring out how to run a lab is typically very much a trial-and-error process. In order to make this process a little less hit-and-miss, I'll here provide some potentially useful pointers by exploring the way I run my own lab.

*“Few young PIs will realize that getting started is the easy part.”*

## What's This Lab about Then?

My group is into bioinformatics, focusing on the management and analysis of high-throughput proteomics data. We develop new algorithms, end-user-oriented software tools, and we try to shift a few paradigms in the process. We are currently hard at work to repurpose publicly available proteomics data to discover rare but biologically significant translation products and protein modifications, an interesting task that requires in-depth integration and specific customization of a variety of our existing software tools and algorithms.

## A Brief History and Outline of the Group

**Start of the Lab:** October 1, 2009

**Size of the Lab:** 15

**Research Field:** proteomics informatics

The Computational Omics and Systems Biology (CompOmics) group was started in October 2009, when I left my position as PRIDE Group Coordinator at EMBL-EBI in Cambridge, UK for a tenured professor position at Ghent University and a group leader position at VIB, both in Ghent, Belgium. Upon arrival in Ghent, I was lucky enough to have access to two very talented PhD students from the existing proteomics group, and together with two PhD students I was co-supervising abroad, I had access to some initial manpower. I did not receive any actual positions for my group however, nor did I get any tangible seed funding, so I had to start obtaining funding and resources essentially from scratch. Fortunately, I managed to succeed at this, and my group now consists of 15 people: nine PhD students, three postdocs, two software developers, and me. Five of the PhD students are day-to-day supervised by one of the postdocs. Four of the PhD students in the group also have formal co-supervisors from other groups; this provides a very useful way to create direct lines of contact to colleagues in the department, in other faculties, and even at other institutes. The two software developers work very closely with the PhD students and postdocs, and they each already have several published papers to their name. And since several PhD students are also actively developing software, the distinction between the various team members is mostly an administrative one.

## It's All about Communication, Even in Bioinformatics!

Communication is the magic ingredient that can knit together an efficient team from a collection of talented individuals. Getting people to communicate can be hard, however. The most important thing is that people need to be able to talk to each other whenever they want to. I therefore made sure that all members in my team are located in one shared office, and that there are plenty of small and portable stools standing around so anyone can sit at a colleague's desk for a while, or organize an impromptu meeting. I also actively seed communication by talking to my team often, and not just about work. Cracking some jokes or telling some funny stories is a great way to get people to feel relaxed and comfortable in the group, and will entice them to do the same, which will in turn form the basic social fabric of your team. People need to feel relaxed about working with their colleagues, and nothing makes people feel more at home than open and spontaneous communication. I also cherish the resulting feeling of empowerment and involvement that my team enjoys, allowing them to offer new ideas and honest feedback to each other and to me. Speaking of feedback, I make a point of providing constant feedback to my team, and that includes a lot of positive feedback as well. It is strange that a simple “job well done, I'm very happy” compliment is sometimes perceived as much harder to give than a severe reprimand, yet in my experience (both from the compliment-receiving as well as from the compliment-giving side) it is precisely the good feedback that people thrive on.

*“People need to be able to talk to each other whenever they want to.”*

A shared Google calendar for the group keeps track of people's absences and holidays, allowing anyone to quickly check everyone else's availability. For work planning, we have recently adopted Trello to great effect, along with typical Agile development practices such as frequent and focused micro-meetings to discuss progress and issues. The few PhD students I co-supervise in labs abroad are kept in constant contact with the local people in Ghent through Skype, where the ability to create “special interest” Skype groups to serve as *ad hoc* private chat channels has proven itself a great means of quickly disseminating news, updates, bugs, or questions within the relevant subpopulation. Such continuous and fluid communication is complemented with a weekly meeting where a randomly selected person (dice are great tools here) delivers an unprepared chalk talk about one subject (of their own choosing) that they have been working on, with input and questions from the rest of the group. I originally planned these weekly meetings on Friday afternoon, but I have moved them to Wednesday mornings since Fridays didn't work very well—any great ideas that come out are likely put away until after the weekend, which is decidedly suboptimal. A more infrequent, but highly successful, way to create an effective esprit de corps is to organize team-building events. I always try to go for decidedly mellow activities that allow people to interact in a relaxed atmosphere outside of the office environment. Bouldering, a highly accessible form of indoor climbing, was one of the best such outings so far.

*“A randomly selected person (dice are great tools here) delivers an unprepared chalk talk.”*

## Handling Trouble in Paradise

One of the toughest tasks for any manager is to handle issues in the team. Here, I found that it pays to be alert and to preempt issues before they grow. Here again, communication is important. By constantly interacting with the members in my group, and by keeping an eye out for signs of friction, I try to detect any problems very quickly. The atmosphere of complete openness that I cultivate as much as possible in the team has also proven very useful, as the members in my team have so far not had any qualms about actively bringing potential issues to my attention, ranging from reminding people to do their dishes, over better ways of managing the resources on our local cluster, to authorship concerns on manuscripts. Issues are bound to arise occasionally in even the best of teams, but when handled quickly and decisively, I have found there is little chance of any issue morphing into open conflict. The good thing about spotting conflicts early is that you can still discuss them openly and informally, and that there is no need to get quarreling parties together for formal, supervised bilateral communication in an enclosed office—a condition I would already consider seriously escalated. When a problem touches upon a more personal issue however, a one-on-one chat about it in private is the default mode of action. In such cases, I do try to use such a private conversation for more than just addressing the problem at hand though, to give the team member in question a correctly nuanced picture of their overall performance that includes positive aspects and appreciation.

*“The atmosphere of complete openness that I cultivate as much as possible in the team has also proven very useful.”*

## Group Empowerment

The main vision underlying my management strategy is team empowerment. I want everyone in my team to realize that they really are the heart and soul of the lab, that they can contribute to the direction and planning of the work, and that their ideas and their efforts make or break the performance of the group. I therefore listen to anyone with an idea or a comment (be it positive or critical), and I actively solicit the team's opinions on important decisions. Perhaps most notable, job interviews in my lab take place in front (or rather, in the middle) of the whole group, which can sound intimidating at first, but usually works out very well in practice as we try to make applicants feel at ease with a few jokes and some chitchat before the interview begins. After the interview, the group as a whole makes the decision to hire (or not hire) the applicant, taking into account their scientific capabilities as well as their personality and how they'd fit in the group. As a result, newcomers are immediately considered part of the team, since the team decided to hire them. This also implicitly ensures that new hires will fit in nicely with the existing group: an important safeguard since even a single round peg in a square hole can wreak havoc on an otherwise excellent group dynamic. I am happy to say that this hiring strategy has paid off handsomely over the years, and has led to a very close-knit group of enthusiastic and mutually supportive people that I am proud to call my team.

*“The group as a whole makes the decision to hire (or not hire) the applicant.”*

## Solicit Advice—It Tends to Be Free!

*“Keep a mental or even a written list of management practices you encounter.”*

Communication with and within my team has been crucial to keeping everything on the rails, but communication outside of the team has taught me most of the successful tricks I use for this. Indeed, I've had so much useful feedback from so many people over the years that I'll freely admit to having stolen most of the good ideas presented above from others. Regardless of the stage of your career, I can therefore recommend keeping a mental or even a written list of management practices you encounter, either directly or indirectly, and that strike you as either particularly useful, or downright inept. Indeed, you can learn as much from someone else's mistakes as you can from their successes! In our science, we constantly build on the experiences and genius of others, so why should we be any different when it comes to the management of our lab?

